# Nanoprinting organic molecules at the quantum level

**DOI:** 10.1038/s41467-019-09877-5

**Published:** 2019-04-23

**Authors:** Claudio U. Hail, Christian Höller, Korenobu Matsuzaki, Patrik Rohner, Jan Renger, Vahid Sandoghdar, Dimos Poulikakos, Hadi Eghlidi

**Affiliations:** 1 Laboratory of Thermodynamics in Emerging Technologies, ETH Zürich, Sonneggstrasse 3, 8092 Zürich, Switzerland; 2 Max Planck Institute for the Science of Light, Staudtstr. 2, 91058 Erlangen, Germany

**Keywords:** Surface patterning, Nanophotonics and plasmonics

## Abstract

Organic compounds present a powerful platform for nanotechnological applications. In particular, molecules suitable for optical functionalities such as single photon generation and energy transfer have great promise for complex nanophotonic circuitry due to their large variety of spectral properties, efficient absorption and emission, and ease of synthesis. Optimal integration, however, calls for control over position and orientation of individual molecules. While various methods have been explored for reaching this regime in the past, none satisfies requirements necessary for practical applications. Here, we present direct non-contact electrohydrodynamic nanoprinting of a countable number of photostable and oriented molecules in a nanocrystal host with subwavelength positioning accuracy. We demonstrate the power of our approach by writing arbitrary patterns and controlled coupling of single molecules to the near field of optical nanostructures. Placement precision, high yield and fabrication facility of our method open many doors for the realization of novel nanophotonic devices.

## Abstract (web summary)

Integration of emitters for nanophotonic applications requires precise control over their position and orientation. Here, Hail et al. demonstrate the positioning of a single and a small number of oriented molecules with subwavelength accuracy bu direct non-contact electrohydrodynamic nanoprinting.

## Introduction

The interaction of light and matter is usually described by the Hamiltonian $\hat {\cal{H}} = {\hat{\mathbf{E}}} \cdot {\hat{\mathbf{D}}}$, where the ${\hat{\mathbf{E}}}$ and ${\hat{\mathbf{D}}}$ operators denote the electric field of light and the emitter dipole moment, respectively. Contrary to the simplistic considerations of university textbooks, which consider linearly polarized electromagnetic plane waves, ${\hat{\mathbf{E}}}$ has a very strong spatial and polarization distribution in nanophotonic applications. For example, the evanescent fields outside a microresonator or plasmonic nanostructure decay within about 10–100 nm and could rapidly change their polarization. Thus, an effective coupling between a quantum emitter and a photon demands nanometer accuracy in positioning the former with respect to the structure of interest that supports the latter. This requirement becomes more stringent if one aims to scale things up and exploit collective effects among several emitters and photons^1,2^.

Despite some interesting recent progress^3–9^, precise and deterministic integration of quantum emitters into optical systems remains a challenge. For inorganic materials, techniques such as molecular beam epitaxy, lithography, etching and ion implantation have been employed^10–15^. However, a universal and flexible solution is still not available, and in particular, the existing methods are not easily compatible with organic emitters. In this work, we show that the nanodripping mode of electrohydrodynamic printing^16,17^ enables deposition of organic dye molecules at will and close to optical nanostructures, with subwavelength accuracy, down to the single molecule level, and with well-defined orientation.

## Results

### Single molecule nanoprinting process

**Fig. 1:**
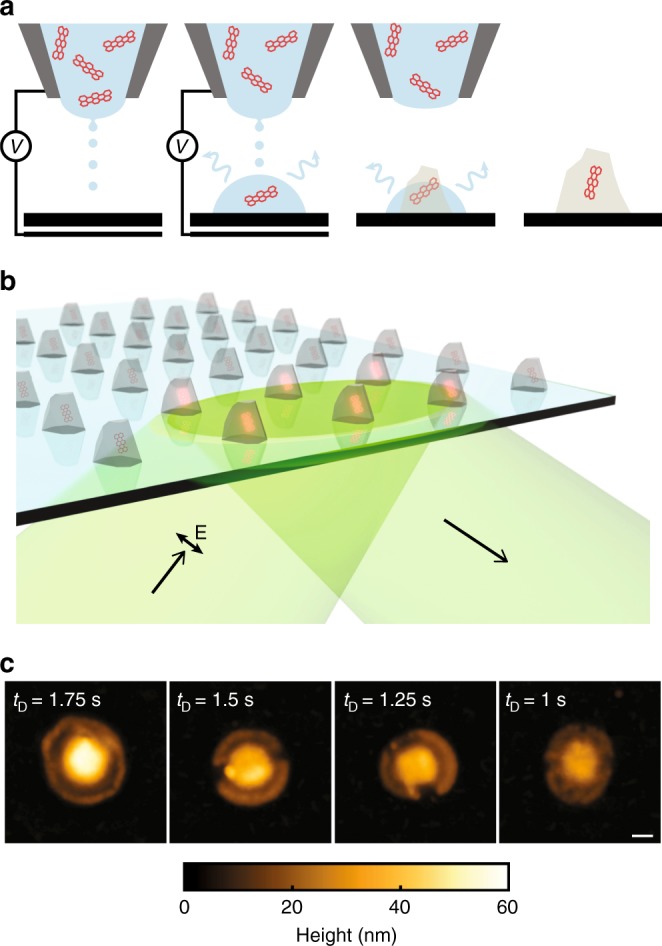
On-demand electrohydrodynamic printing of dye molecules in a host crystal. **a** Schematic representation of the printing process of the host material with embedded fluorescent molecules, showing sequentially the process of dropwise deposition, solvent evaporation and crystallization, capturing a single molecule in the crystallized deposit. A detailed schematic of the experimental setup is provided in the Supplementary Fig. 1. The drawing is not to scale. **b** A schematic representation of a printed array of nanocrystals with embedded fluorescent molecules and illumination direction and polarization. **c** Atomic force microscopy (AFM) images of four printed pT nanocrystals with varying deposition time *t*_D_. The scale bar is 200 nm

We use a gold-coated pulled glass micropipette as printing nozzle to dispense ink onto a substrate (Fig. 1a). Controllable sequences of ink droplets are ejected from the nozzle by applying a DC voltage between the nozzle and a back electrode below the substrate (see Methods section). Electrostatic autofocusing of the ejected sub-attoliter sized droplets of ink ensures a precise placement of the printed material^16^. Printing at any desired location on the substrate can be achieved by moving it laterally relative to the printing nozzle.

The ink used in this work was obtained by dissolving terrylene and *para*-terphenyl (pT) in small concentrations in 1,2,4-trichlorobenzene (see Methods section). Here, pT is to act as the nanoscopic host for a small number of terrylene molecules. *Para*-terphenyl is known to form crystalline matrices via sublimation (below its melting temperature of 487 K)^18^ or spin coating^19^. As we discuss below, we find that our printed nanostructures are also crystalline. This is achieved by the slow evaporation of the ink solvent, which is controlled by actively cooling the receiver substrate to a temperature close to the solvent melting point with a Peltier element. The slow evaporation of the solvent on the substrate is essential for achieving high crystallinity, in contrast to our previous nanoprinting work, where fast evaporation was necessary to avoid liquid accumulation^16^.

Figure 1b schematically shows a printed array of pT nanocrystals. The amount of the printed pT is controlled by the number of nanodrops deposited at a fixed location, the pT concentration in the ink, or by regulating the mass flow rate with the DC voltage amplitude. By optimizing both voltage amplitude and deposition time for a fixed material concentration, the lateral and vertical dimensions of the printed pT nanocrystals can reach down to 220 nm and 30 nm, respectively. Atomic force microscopy scans in Fig. 1c show the effect of the deposition time on the nanocrystal size. We note that for a volatile organic host material such as pT, sublimation in ambient conditions allows further downsizing of the nanocrystal to a few nanometers (see Supplementary Note 1). However, in this work, we avoid this process by protecting the nanocrystals with a thin polymer layer.

### Optical and quantum optical characterization

**Fig. 2:**
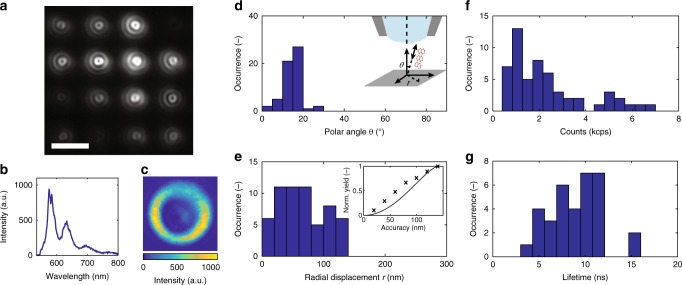
Optical characterization of the printed dye molecules. **a** TIRF microscopy image of an ordered array of printed terrylene molecules embedded in pT nanocrystals on a glass substrate and protected from sublimation with a 100 nm thick poly(vinyl alcohol) layer. The scale bar is 2 μm. (see Supplementary Fig. 5 for a schematic of the optical setup). The neighboring nanocrystals can be placed at a distance down to 0.65 μm (see Supplementary Fig. 6). **b** Fluorescence spectrum of a printed terrylene molecule. **c** Back focal plane image of a printed terrylene molecule oriented with a polar angle of *θ* = 16.7 ± 0.5° to the substrate. **d** Histogram of angular orientation of the molecule embedded in the host crystal with respect to the surface normal direction as shown in the inset. **e** Histogram of the in-plane radial distribution of molecules around the mean position of the molecules, which ideally corresponds to the printing nozzle axis. The inset shows the measured normalized yield (crosses, normalized to the yield of achieving one molecule per printed spot) for a varying in-plane positioning accuracy compared to a numerical calculation (solid line) of random placement in a hemispherical, printed crystal with the same size. **f** Histogram of fluorescence count rates of printed single molecules. **g** Histogram of fluorescence lifetime fitted from continuous wave anti-bunching curves. The mean confidence interval of the fits is ± 0.7 ns. The inset in **d** is not shown to scale. The fluorescence image of the entire 10 × 10 array from **a**, and for the extracted data in **d**, **e** is shown in Supplementary Fig. 4. **f**, **g** are obtained at constant illumination of ~5000 W cm^−2^

By seeding the ink with a low concentration of terrylene as fluorescent dye, a small number of terrylene molecules can be deposited in a controlled fashion at specific positions, whereby the average number of molecules per printed nanocrystal can be tuned by adjusting the amount of the deposited ink or the concentration of the dye molecules. Terrylene in pT is a well-known system used for a variety of quantum optical studies^19–23^. In particular, it is known that terrylene embeds inside pT at well-defined sites and orientation^19,24^. Figure 2a shows a fluorescence image from an ordered array of printed spots of host material with embedded fluorescent molecules at a separation of 2 μm measured on a total internal reflection fluorescence (TIRF) microscope.

The printed nanocrystals were protected from sublimation with a 100 nm thick poly(vinyl alcohol) (PVA) layer, which also helped to increase the average photostability of terrylene molecules. The majority of the printed molecules exhibited stable fluorescence over several minutes to hours or even days at ambient conditions, while a small fraction (less than 25%) bleached either immediately or after a few minutes of illumination at about 100 W cm^−2^ (See Supplementary Fig. 2 for more details). The observed photostability indicates the high quality of the formed host crystal and the seamless integration of the molecules in it^19^. We point out that the printed molecules could also withstand high illumination intensities up to saturation levels (see Supplementary Fig. 3).

The emission spectrum of a single printed molecule is shown in Fig. 2b with little to no background signal in agreement with the previously reported spectra of terrylene^22^. The back-focal plane image of the same molecule shown in Fig. 2c presents its angular distribution of emission corresponding to a dipole moment oriented at a small angle (here *θ* = 16.7 ± 0.5°) with respect to the normal to the substrate^19^. The histogram in Fig. 2d displays the distribution of the dipole orientations of the photostable (after few minutes) printed molecules from a 10 × 10 array (see Supplementary Fig. 4 for fluorescence image). This well-defined orientation of the printed molecules can also be directly recognized from the doughnut-shaped fluorescence images in Fig. 2a, which indicate a molecular emission dipole moment almost perpendicular to the substrate surface^19^ and are characteristic of terrylene in pT. This characteristic orientation of terrylene embedded in pT is a good indicator for the crystallinity of pT. The angular orientation of molecules in a spin-coated crystalline pT matrix shows a very similar dipole orientation distribution to that of molecules in nanoprinted pT. This is a strong indirect indication of the good crystallinity of the nanoprinted pT (see Supplementary Note 2).

The histogram in Fig. 2e shows the radial distribution of the photostable molecules with respect to the mean molecule position determined for the array of 10 × 10 printed spots. 43% of the molecules are found within a distance of 50 nm, and 90% within 120 nm from the mean position. In the vertical direction, the molecules are confined by the height of the nanocrystals, which can be as small as 30 nm (see Fig. 1c). From these results, the yield for a given accuracy of in-plane positioning can be calculated (see inset Fig. 2e). In-plane accuracies of down to ±20 nm can be obtained with a 10% normalized yield.

Figure 2f shows the distribution of fluorescence count rates measured from single printed molecules excited with the same illumination intensity. Below, we will elaborate on rigorous tests for identifying single molecules. The variation observed in Fig. 2f results from differences in quantum efficiency of the molecules^23^, molecular dipole orientation, or location of the molecule within the crystal. To examine the quantum optical properties of the printed single molecules further, we also performed fluorescence lifetime measurements using a Hanbury–Brown and Twiss (HBT) setup^25^. Figure 2g displays the distribution of the fluorescence lifetimes. While the lower value in the range of 4 ns corresponds to the expected fluorescence lifetime of terrylene in bulk pT, longer lifetimes are characteristic of molecules close to an interface and oriented normal to it^26^. In our case, we expect the subwavelength lateral dimension of the nanocrystal to also contribute to the longer lifetimes^27,28^.

**Fig. 3:**
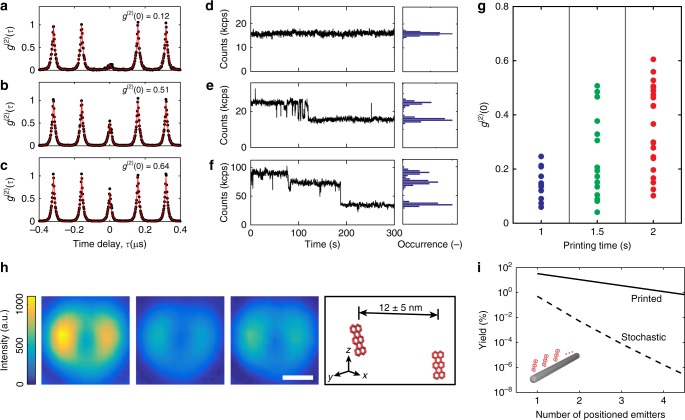
Quantum optical characterization of the printed fluorescent molecules. Measured normalized second order correlation function of one (**a**), two (**b**), and three (**c**) dye molecules embedded in a host crystal. Fluorescence time traces and corresponding histograms showing a stable photon count of one printed molecule (**d**), a one-step bleaching event of two (**e**), and a two-step bleaching event of three molecules (**f)**. **g** Measured values of *g*^(2)^(0) of photostable molecules with increasing deposition times. **h** Two molecules with an in-plane separation of 12 ± 5 nm in a single printed nanocrystal. The first panel (left) shows a fluorescence image of both molecules, the second shows the remaining molecule after the other has bleached. The third panel shows the image of the photobleached molecule obtained by subtracting the intensities in panel two from panel one. The scale bar is 200 nm. The rightmost panel shows a schematic of the two molecules in close proximity, based on the position and orientation from the localization analysis (the molecule size is enlarged by approximately 3.5 times for better visibility). See Supplementary Fig. 7 for fluorescence count rates and *g*^(2)^(*τ*) before and after photobleaching. **i** Probability analysis for printing vs. stochastically placing quantum emitters in specified areas. The expected yields are shown for the example of placing a number of emitters in the vicinity of a 10 μm long waveguide structure as shown in the inset (See Supplementary Note 3 for details on the calculation). Lines are drawn as a guide to the eye. In **a**–**f** pulsed excitation at 6.25 MHz was used and in **g** and **h** continuous wave excitation was used

To prove that we reach the fundamental quantized limit of printing matter, namely single molecules, we performed HBT experiments and analyzed the intensity time traces to deduce the second order correlation function *g*^(2)^(*τ*). Figure 3a displays the outcome with *g*^(2)^(0) = 0.12, which is well below the limit of 0.5 as the threshold for having fewer than two molecules according to $g^{(2)}(0) = 1 - \frac{1}{m}$, where *m* denotes the number of quantum emitters^25^. The steady intensity trace in Fig. 3d supports this finding and shows the photostability of the molecule.

The ability to deposit a single fluorescent molecule at a well-defined position is a long-sought and significant step towards nanophotonic devices, e.g., for single photon generation. However, as quantum technologies emerge, there will be an increasing demand for controlled realization of few-photon and few-emitter systems for scaling purposes and more sophisticated quantum state generations^2,29^. We now show that by varying the deposition time, we are able to adjust the statistical mean of the number of molecules per printed nanocrystal and, therefore, deposit multiples of molecules in close proximity. Figure 3b, c present the measured *g*^(2)^(*τ*) from two representative printed spots, reaching *g*^(2)^(0) = 0.51 and *g*^(2)^(0) = 0.64, indicating two and three printed molecules, respectively. Accordingly, the fluorescence time traces of the printed molecules in Fig. 3e, f display one and two-step bleaching events.

To examine the dependence of the printed molecule number on the deposition time, in Fig. 3g we present *g*^(2)^(0) values for many nanocrystals. The observed trend of increasing *g*^(2)^(0) with longer deposition times agrees with the expected increase in the mean number of printed molecules per spot, demonstrating that the mean number of molecules can be controlled. We note that measurement non-idealities such as the background signal, noise, varying quantum efficiency of emitters and blinking can result in deviations in the *g*^(2)^(0) value.

Using stepwise bleaching localization microscopy^30^, in Fig. 3h we resolve two molecules in the same nanocrystal at a lateral separation of 12 ± 5 nm (see Supplementary Fig. 7, for more details). This close proximity can be used to couple two or more molecules via near-field dipole-dipole interaction at much greater yield than the stochastic approach exploited previously^31^. Assuming a random distribution in a rectangular cuboid crystal, the normalized yield for obtaining a certain separation between two molecules can be estimated (see Supplementary Fig. 8). By reducing the lateral dimension of the crystal, normalized yields exceeding 34% may be reached for separations as small as 12 nm.

A critical issue in many nano-optical experiments is to find a balance between a high success rate in studying a controlled number of emitters versus the risk of a high background fluorescence from unwanted emitters^5^. Figure 3i compares the expected yield of placing single molecules at specified positions by printing with that of stochastic distribution for the example of a nanowire. In this case, we obtain the probabilities of 33% for coupling a single emitter or 4% for simultaneously coupling three emitters to the nanowire at specified locations (see Supplementary Note 3). These success rates are orders of magnitude higher than the values of 0.5% and 8 × 10^−5^% expected for randomly placing molecules.

### Coupling molecules to nanophotonic structures

**Fig. 4:**
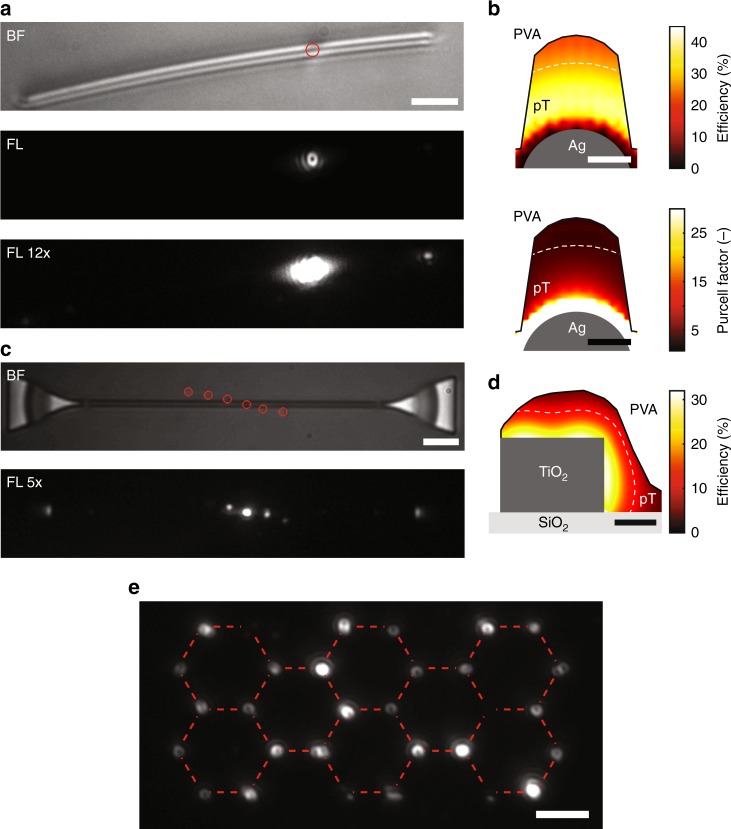
On-demand printing of molecules near nanophotonic structures and in arbitrary patterns. **a** Bright-field (BF) and fluorescence (FL) image of a molecule printed on a single crystalline silver nanowire. The same fluorescence image is shown with the intensities scaled by 12× (FL 12×), where the out-coupled light is clearly visible. The faint fluorescence spot at the left end of the nanowire is due to coupling of the photons to the opposite direction and its lower intensity is attributed to larger propagation losses in that direction. The scale bar is 2 μm. **b** Simulated coupling efficiency and Purcell factor depending on the location of the molecule with respect to the silver nanowire. The dashed white lines correspond to a coupling efficiency of 30%. Calculations were performed for a vertically oriented dipole inside the printed pT nanocrystal (with its border shown by the solid black lines) and a nanowire of 80 nm diameter. The scale bars are 30 nm. **c** Bright-field (BF) and fluorescence (FL 5×) images of molecules printed on a TiO_2_ waveguide with focused laser illumination. Fluorescence images are scaled by 5x in intensity with respect to full scale for better visibility. The red circles mark the location of the printed nanocrystals. The scale bar is 4 μm. **d** Simulated coupling efficiency depending on the location of the molecule with respect to the TiO_2_ waveguide. The white dashed line represents the possible location of the molecule based on the measured coupling efficiency of 12%. Calculations were performed for a vertically oriented dipole inside the printed pT nanocrystal (with its border shown by the solid black lines). The scale bar is 100 nm. **e** Fluorescence image of printed terrylene molecules on a glass substrate composing collectively the molecular structure of terrylene. The dashed, eye-guiding line aids the recognition of the outline of the molecular structure of terrylene. The scale bar is 2 μm

We now present two examples of applications for coupling molecules to photonic nanostructures. Figure 4a displays the bright-field image of a single crystalline silver nanowire with a diameter of approximately 80 nm. In Fig. 4a, we show the fluorescence image of a single terrylene molecule printed on the nanowire imaged with a tightly focused excitation beam. Antibunching measurements, fluorescence spectrum, and the doughnut shaped image of the molecule clearly point to an oriented single terrylene molecule. To investigate the coupling of the molecular emission to the nanowire, we recorded the fluorescence at the end of the nanowire, as shown in Fig. 4a. Attribution of this light to the molecule was verified by the correlated intermittency of the molecular fluorescence and the out-coupled light, and by recording an image after deliberate photobleaching. Furthermore, we observed a significant shortening of the mean lifetime of the molecules coupled to nanowires. A comparison of the fluorescence intensities suggests an apparent coupling efficiency of up to 6.2% into the surface plasmon mode, which, in fact, would be up to 3–5 times larger (i.e., 18–30%) when accounting for ohmic losses in the nanowire as done in previous studies^32^. Figure 4b shows the simulated coupling efficiency and Purcell factor for different positions of the molecule in the host crystal and close to the silver nanowire. Simulations are shown only inside the pT crystal (within the border shown by the solid lines) and were performed for a dipole moment vertical to the substrate and a nanowire of 80 nm in diameter. Both the size of the nanowire and the printed host nanocrystal were measured from AFM scans on the PVA-coated structure. More information about the simulations is available in the Methods section. Based on these simulations a distance of 45–70 nm between the molecule and the nanowire surface is estimated. For more details on the discussion above, see Supplementary Note 4.

As a second example, we coupled a terrylene molecule to a subwavelength dielectric waveguide made of TiO_2_, which offers an efficient avenue for minimizing propagation losses associated with plasmonic structures. Figure 4c shows a bright-field image of a waveguide, which is 200 nm wide and 180 nm high with integrated grating couplers, and a fluorescence image of several printed molecules in its vicinity under focused illumination. The excitation light triggers a photon emission, which partially couples to the waveguide mode and is out-coupled at both ends. Here too, the signal at the grating couplers vanishes after the photobleaching of the molecule, confirming the coupling of the molecule emission to the waveguide mode (See Supplementary Note 5). From the measurement, a coupling efficiency of 12% is estimated. Figure 4d shows the simulated coupling efficiency for different positions of the molecule inside the host crystal with respect to the TiO_2_ waveguide. Based on these simulations, the distance between the molecule and the waveguide surface is estimated to be 65 ± 5 nm.

We find that for the case of the silver nanowire an uncertainty in positioning of the molecule within the crystal shown in Fig. 4b can result in a coupling efficiency variation in the range of 20–40%. For the TiO_2_ ridge waveguide, we expect a variation of 6–32% in the coupling efficiency. This indicates that, for a nanoscopic crystal printed on top of the waveguides, the molecule would be coupled to the waveguides with high reproducibility.

Furthermore, our open-atmosphere, maskless, fabrication method is capable of placing molecules in any arbitrary pattern, such as the chemical structure of a terrylene molecule shown in Fig. 4e.

## Discussion

We have presented a method for versatile printing of a countable number of terrylene molecules within a nanoscopic volume on different substrates (metalic, inorganic, flexible; see also Supplementary Fig. 9) and structures. This work represents a remarkably higher level of control and scalability in placement of single quantum emitters, in comparison to previous reports, where deposition of quantum emitters in large numbers^33–36^, or uncontrollably at random^37^ was demonstrated. A comparison of different methods for single quantum emitter placement is given in Supplementary Table 1. The printing performance can be further improved in several ways. The positioning uncertainty of the deposited molecules can be reduced by lowering the crystal size. This can be achieved by adjusting the ink concentration or by means of post-sublimation. Moreover, one can improve the yields close to 100% by implementing a monitoring mechanism to determine the number of photostable molecules, e.g., via in-situ fluorescence measurement while printing, and repeating the printing step. Furthermore, the method can be readily extended to other molecule-host systems^38^ and be combined with printing of nanostructures such as optical nanoantennas or nanowires^16^ in a single platform.

Our demonstrated achievements open the doors for the efficient production of nanophotonic devices based on single molecules, such as single-photon guns^23^. In particular, our printing method can be used for coupling a larger number of molecules along an optical circuit or extended structure, e.g., for the realization of devices for quantum information processing^39^, polaritonic states^2^ or quantum metamaterials and networks^29^. Furthermore, one can place several molecules in subwavelength vicinity of each other for achieving controlled coherent coupling^31^ and for the efficient generation of multiphoton states when coupled to suitable optical antennas^31,32^. Given the existing crystallinity of the pT matrix and possible further improvement through post-annealing^40^, we expect good coherence properties of the printed terrylene molecules at cryogenic temperatures. In all the applications mentioned above, the placement accuracy, flexibility of combination with different materials and the simplicity of our method will be an invaluable asset.

## Methods

### Nanoprinting

The nanocrystals are deposited by electrohydrodynamic nanoprinting, where desired sequences of nanodroplets are deposited sequentially on a substrate by applying a DC voltage between the printing nozzle and a back electrode below the substrate, and realizing and exploiting a rapid nanodripping droplet generation mode^16,17^. The printing nozzle consists of a borosilicate capillary (*World Precision Instruments TW100-4*) that is pulled (*Sutter Model P-97)* to nozzle openings ranging between 1 and 2 μm which is subsequently coated with 5 nm titanium for adhesion and 50 nm gold by electron beam evaporation. The ink consists of a 1:57 mixture of 0.06% saturated terrylene and pT (2.3 mg ml^−1^) each dissolved in 1,2,4-trichlorobenzene. For printing, samples were placed on a Peltier cooled sample holder (sample temperature 16.5 °C), which includes an ITO-coated glass piece as back electrode below the substrate. With the backfilled nozzle in close proximity (4–5 μm) of the substrate surface and applying a DC voltage (200–230 V) for a specified deposition time ranging between 1 and 3 s, nanodroplets are ejected from the nozzle onto the substrate. The ink composition, sample temperature and the ejection rate of nanodroplets are optimized such that a nanoscale pool of ink accumulates on the substrate and by slow evaporation of the ink solvent, a material with high crystallinity is formed, ensuring the seamless integration of terrylene into the pT host. To accurately move the sample in the in-plane and out-of-plane directions, while the nozzle is held at a fixed position, a piezo and a motorized micro translation stage were used. Optical access during printing is provided by an inverted scanning interference microscope and a side view with a long working distance objective. Details on the main features of the experimental setup for electrohydrodynamic printing can be found elsewhere^16^.

### Sample fabrication

The structures were fabricated on a borosilicate glass coverslip that was extensively cleaned before printing by sonication in acetone, isopropyl alcohol (IPA) and deionized water for 20 min each and then by O_2_ plasma cleaning. After printing, the nanocrystals were protected from sublimation by spin-coating a 100 nm thick layer of poly(vinyl alcohol). For printing on silver nanowires, nanowires (average diameter 130 nm) in isopropyl alcohol solution were spin-coated onto a clean glass coverslip. Molecules with their host crystal were printed directly onto the wires by localizing the nanowire and the printing nozzle through the bottom microscope objective. Dielectric waveguides were written by electron beam lithography in negative tone resist and reactive ion etching was used to transfer the pattern into a 180 nm thick TiO_2_ layer deposited on a glass substrate.

### Measurements

The samples were analyzed on a home-built total internal reflection fluorescence microscope with an oil immersion objective (NA = 1.4), continuous wave illumination (wavelength 532 nm) and an EMCCD camera (*Andor iXon 888*). Supplementary Fig. 5 shows the experimental setup. All measurements have been carried out at room temperature and ambient conditions. The position and dipole orientation of the molecules were determined by comparing the measured images to the theoretically calculated point spread function of an oriented dipole at an interface and applying a maximum likelihood estimate^41^ for each printed spot. Photon correlation measurements were performed with pulsed (6.25 MHz) or continuous wave excitation (wavelength 532 nm) with a Hanbury–Brown and Twiss setup with two single photon detectors (*MPD PD-050-CTB* and *ID Quantique ID100-50*). For the measurement of the dielectric waveguides a 100× air objective was used. Atomic force microscopy scans were performed within 6 h after printing.

### Simulations

Numerical simulations of the coupling of molecules to silver nanowires or TiO_2_ waveguides were performed using the finite-difference time-domain method (FDTD). The simulated structures consist of a silver nanowire or a rectangular TiO_2_ waveguide on a glass substrate covered with a 100 nm thick PVA layer. The molecules are modeled as a vertically oriented dipole source. The optical properties of silver nanowires are taken from literature values for polycrystalline silver^42^, the glass substrate is described with a refractive index *n* = 1.53, TiO_2_ with *n* = 2.4, PVA with *n* = 1.48 and pT with *n* = 1.6. Perfectly matched layers are applied as boundary conditions on all sides of the simulated structures. Simulations are stopped when the total power in the simulation domain has decreased below a fraction of 10^−6^ of the total injected power. For the nanowire, the coupling efficiency is determined by integrating the Poynting vector over a cross-sectional area perpendicular to the wire at an axial distance of 3 μm from the dipole along the nanowire, and correcting for the dissipation in the nanowire. The Purcell factor is calculated by dividing the radiated power of the dipole in the presence of the nanowire by the radiated power of the dipole on a substrate embedded in a 100 nm PVA layer. For the TiO_2_ waveguide the coupling efficiency is determined by integrating the Poynting vector over an area perpendicular to the wire at an axial distance of 3 μm from the dipole along the waveguide.

## Supplementary information

Supplementary Information

## Data Availability

The data that support the plots within this paper and other findings of this study are available from the corresponding authors upon reasonable request.
