# Supplementary material for: Nanoprinting organic molecules at the quantum level
Source: Nat Commun. 2019 Apr 23;10:1880. doi: 10.1038/s41467-019-09877-5 (PMC6478689; doi:10.1038/s41467-019-09877-5)
Supplement: Supplementary file 1 — Supplementary Information [file 41467_2019_9877_MOESM1_ESM.pdf]

Supplementary Information: Nanoprinting organic molecules at the quantum level

Hail *et al.*

Supplementary Figures

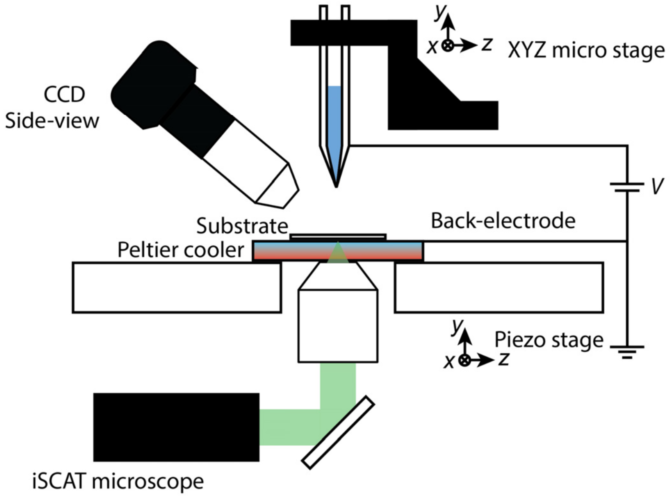

Supplementary Figure 1 | Experimental setup for electrohydrodynamic printing of fluorescent molecules embedded in a host material. For printing, a sample is mounted on a Peltier cooled sample holder that is fixed onto a piezo stage for translating the sample in plane. The pulled gold-coated glass capillary is brought into close proximity (4–5 μm) of the sample with a microstage and is electrically connected to a high voltage amplifier and a signal generator. The observation of the printing process is through the substrate by means of an iSCAT microscope[1] and through a side-view large working distance objective.

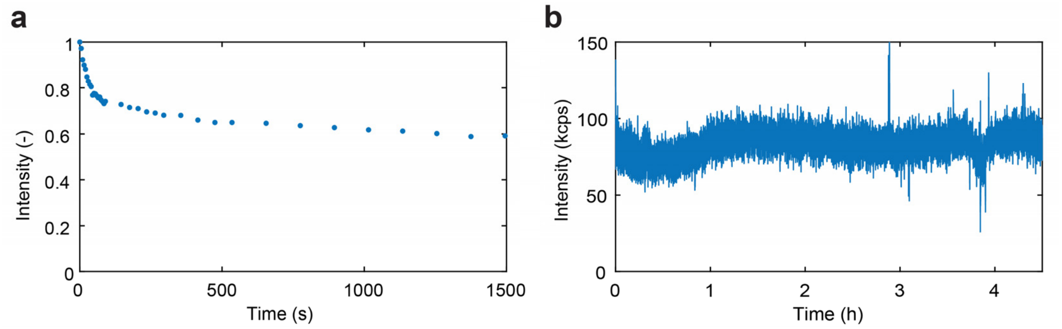

Supplementary Figure 2 | Measured photostability of nanoprinted molecules. a, Fluorescence intensity of a 10x10 array of printed molecules over time under constant continuous wave laser illumination of ~100 W cm^{-2}. **b,** Fluorescence time trace of a molecule exhibiting a stable emission over a time duration of 4.5 h, with pulsed laser excitation and an average illumination intensity of ~300 W cm^{-2}.

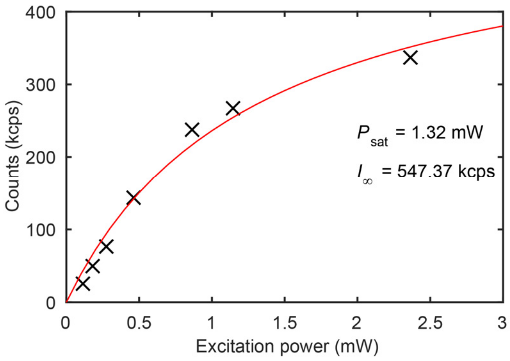

Supplementary Figure 3 | Saturation curve of a printed molecule. Fluorescence saturation curve obtained on a printed molecule with focused continuous wave illumination yielding P_{sat} = 1.32 mW and I_{∞} = 547.37 kcps from fitting the theoretical curve. Here the excitation beam was focused to an area of ~ 1.3 μm × 1.6 μm.

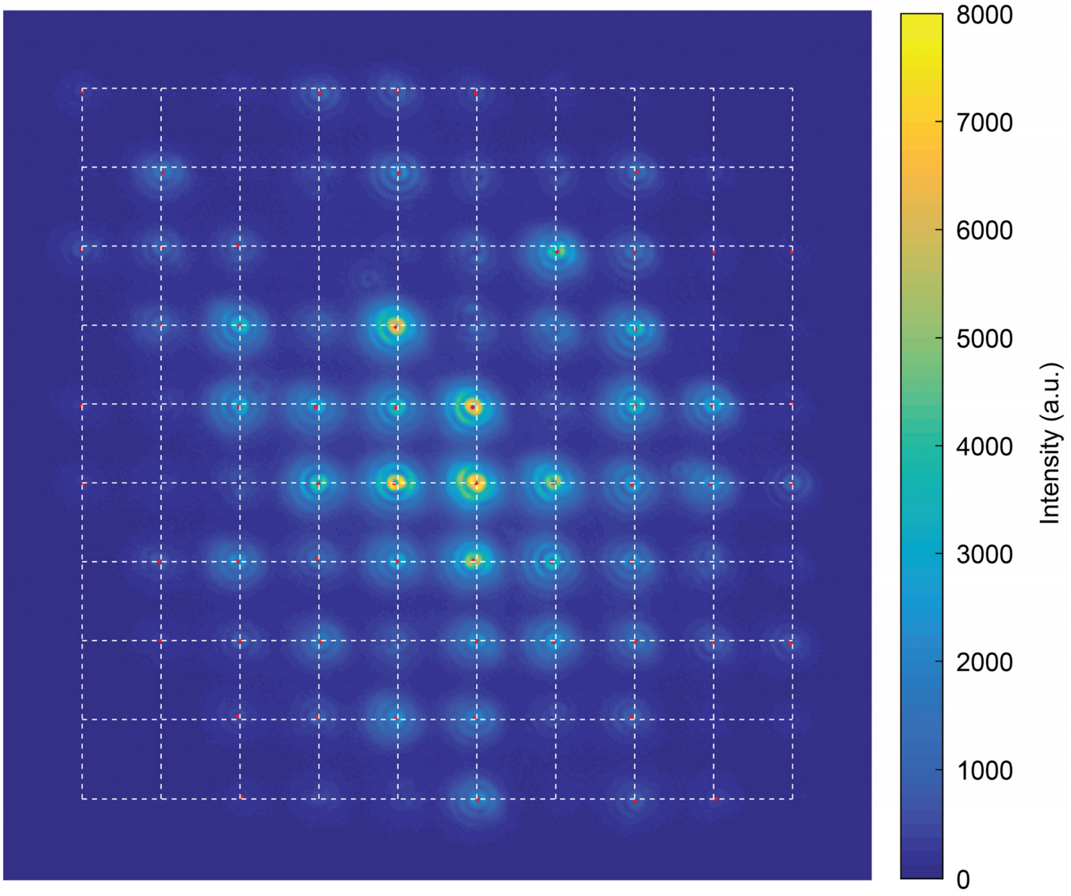

Supplementary Figure 4 | A large 10x10 array of printed molecules for localization. A TIRF image of printed *para*-terphenyl (pT) nanocrystals with embedded terrylene molecules at a separation of 2 μm. The intersections in the white grid show the mean locations of the molecules at a fixed 2 μm separation, corresponding to the intended spot of deposition, and the red dots show the localized positions of the molecules. At sites where no red dot is shown either no molecule or multiple molecule were printed, or the localization analysis yielded errors exceeding 50 nm. These sites are not considered for the analysis shown in Fig. 2. Both position and dipole orientation of the molecules were determined by comparing the measured images to the theoretically calculated point spread function of an oriented dipole at an interface and applying a maximum likelihood estimate[2] for each printed spot.

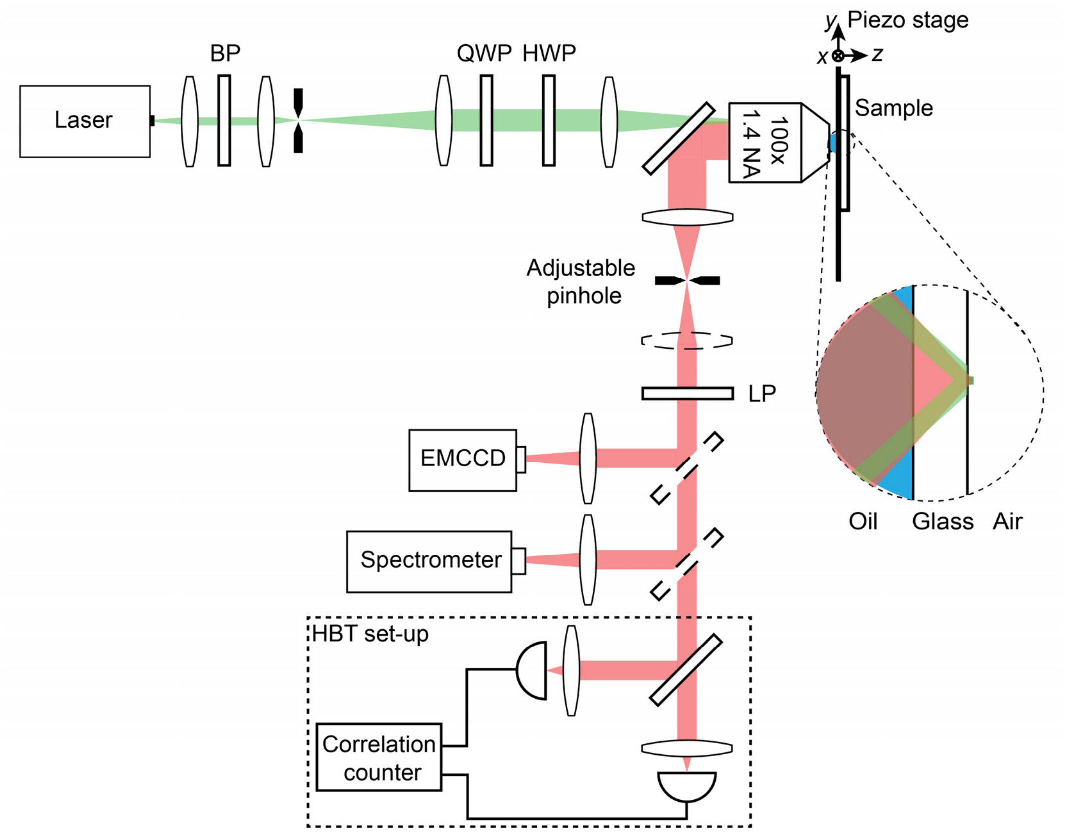

Supplementary Figure 5 | Total internal reflection fluorescence microscope. A quarter wave plate (QWP) and half wave plate (HWP) are used to set the polarization of the excitation beam. A band-pass (BP) filter is used to obtain a spectrally narrow excitation beam, and a long-pass (LP) filter is used in the detection path to reject the excitation beam. Removable lenses or mirrors are drawn with dashed lines. Photon correlation measurements are performed with an HBT setup with two single-photon avalanche diodes connected to a correlation counter. For localizing the two molecules in Fig. 3h the mirror in front of the EMCCD camera is replaced with a beam splitter to allow simultaneous imaging and correlation measurements.

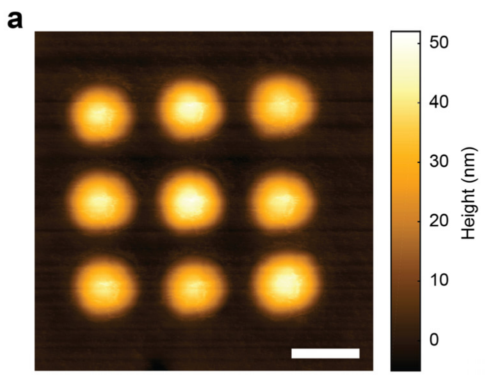

Supplementary Figure 6 | Printed arrays of nanocrystals with embedded molecules with a small pitch. a, An atomic force micrograph of a three by three array of printed nanocrystals with embedded molecules with a separation of 650 nm. The surface is coated with a thin (below 100 nm) PVA layer to protect the nanocrystals from sublimation. **b,** Total internal reflection fluorescence microscopy (TIRF) image of a three by three array of printed nanocrystals with embedded molecules with a separation of 650 nm. The scale bars are 500 nm.

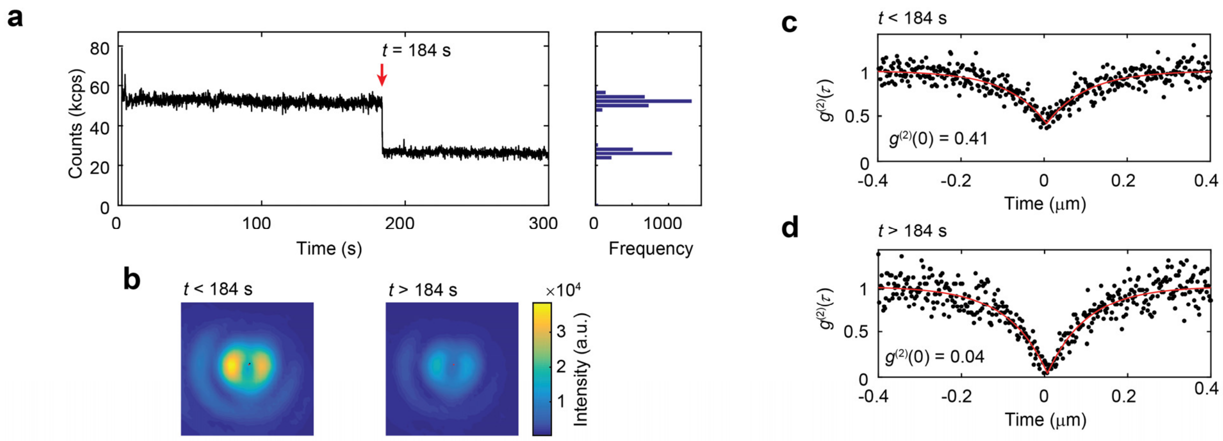

Supplementary Figure 7 | Two closely spaced molecules in a single printed nanocrystal. a, Fluorescence time trace of the two molecules with the photobleaching of one molecule occurring at t = 184 s. **b,** Image of the two molecules acquired at t < 184 s and an image of the remaining molecule acquired at t > 184 s. **c.** Second order correlation function acquired at t < 184 s with $g^{(2)}(0) = 0.41$ indicating the presence of two molecules. **d,** Second order correlation function acquired at t > 184 s with $g^{(2)}(0) = 0.04$ indicating the presence of a single molecule. The red curves in **c** and **d** are single exponential fits to the experimental data.

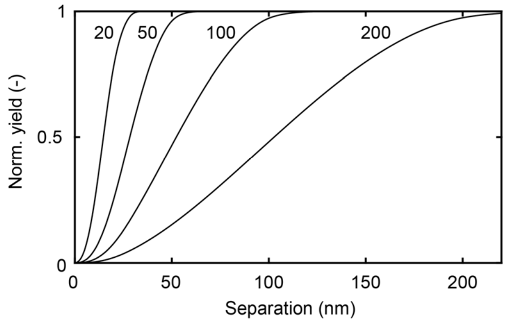

Supplementary Figure 8 | Expected yield for obtaining close separations between two molecules in the same printed crystal. Calculated normalized yield for obtaining a pair of molecules within a desired separation distance in one nanocrystal for varying sizes of the nanocrystal. For the calculation, nanocrystals are assumed to be rectangular cuboids with a height of 30 nm and a varying width and length of *w* = 20, 50, 100 and 200 nm.

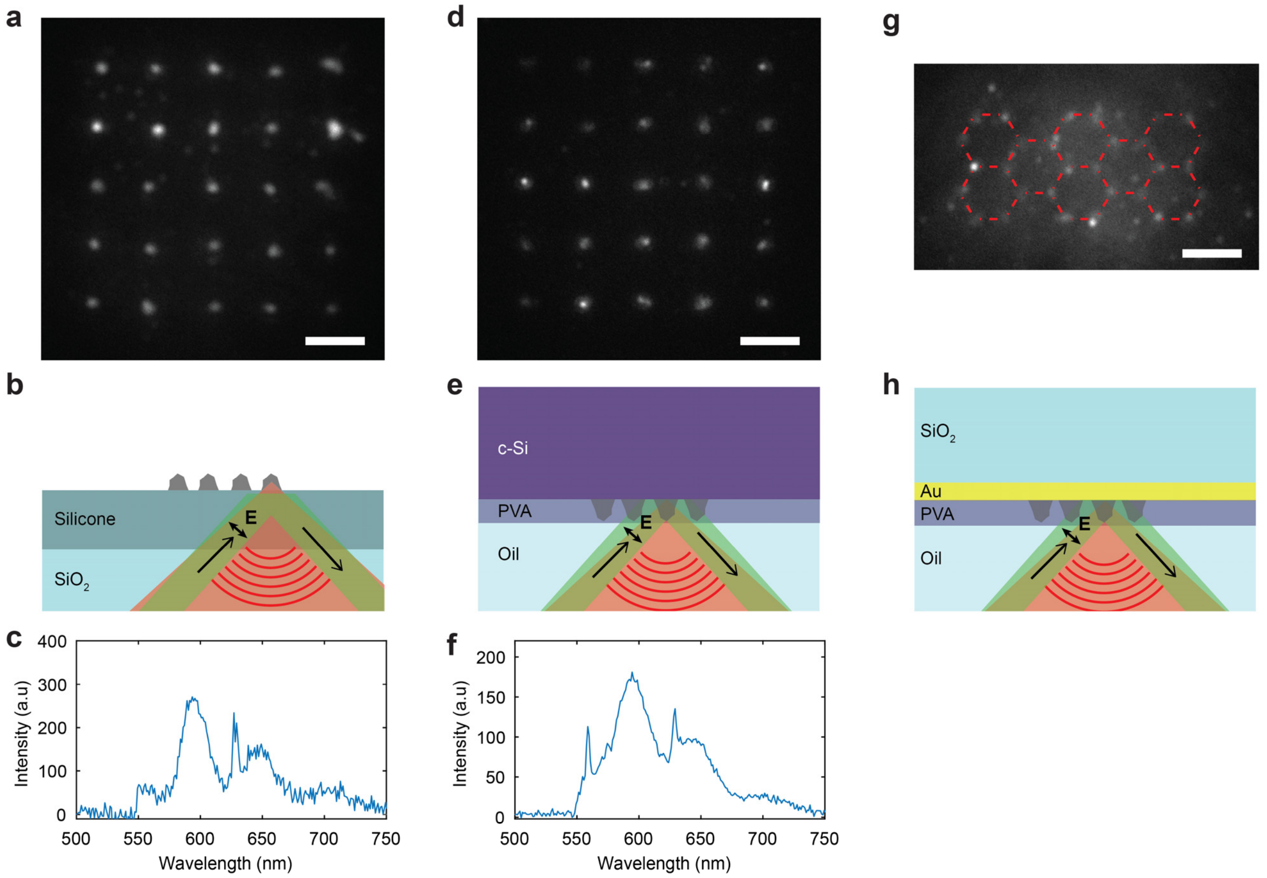

Supplementary Figure 9 | Printing molecules on different substrates. a, Fluorescence image of printed molecules on a highly deformable silicone rubber substrate (thickness 30 μm) on glass. **b,** Schematic of the substrate composition and illumination. **c,** Fluorescence spectrum collected from a printed molecule on the silicone rubber substrate. **d,** Fluorescence image of printed molecules on a silicon substrate. **e,** Schematic of the substrate composition and illumination. **f,** Fluorescence spectrum collected from a printed molecule on the silicon substrate. **g,** Fluorescence image of printed molecules on a 15 nm thick gold layer. **h,** Schematic of the substrate composition and illumination. The scale bars are 3 μm.

Supplementary Tables

Method	Accuracy	Yield	Type of emitters	Coherence properties	Process characteristics
Spin coating	None	-	Colloidal QDs, molecules,	Good at low T	• Single step<br>• Ambient conditions
Lithographic	±10 nm[3]	N.A.	Colloidal QDs	Very low even at low temp, not well characterized	• Multistep
Implantation	±15 nm[4]	37%	NV centers	very low at RT	• Multistep<br>• High vacuum
DNA Origami	<1 nm[5] (a)<br>± 25 nm[6] (b)	85%[6]	Colloidal QDs, molecules	Expected to be very low, not well characterized	• Multistep<br>• High vacuum for lithographic placement on a surface<br>• Molecules with poor photostability
Laser induced NV	± 400 nm[7]	96%[8]	NV centers	Good at low T[7]	• High power laser
Directed QD assembly	± 15 nm[9]	N.A.	Epitaxial QDs	Good at low T	• High vacuum
Nanoprinting (this work)	±120 nm	36%	Molecules	Expected to be good, not characterized yet	• Single step (facile)<br>• Oriented emitters<br>• Ambient Conditions<br>• Highly photostable molecules

Supplementary Table 1. Comparison of different techniques for nanopositioning of organic and inorganic quantum emitters. (a) Resolution with respect to other DNA attached structures in solution. (b) With respect to non-DNA attached structures on a surface. RT: room temperature.

Supplementary Notes

Supplementary Note 1: Nanocrystal sublimation

Using a volatile organic host material, the size of the printed nanocrystal may be reduced further after deposition. Due to the sublimation of the crystal in ambient conditions, the crystal volume shrinks over time (Supplementary Fig. 10). The shrinkage occurs first along a lateral dimension (*x*), then along the perpendicular lateral dimension (*y*) and finally along the vertical dimension (*z*). This may likely be due to the crystal orientation and different sublimation timescales associated with different crystal planes. The sublimation of the crystal allows for further downsizing of the printed nanocrystals, which can then be stopped by protecting the nanocrystals with a thin polymer layer.

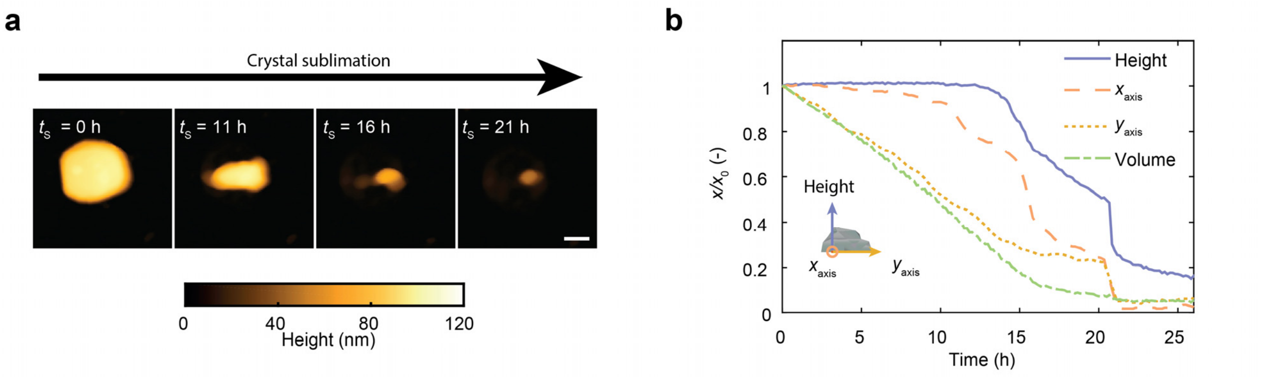

Supplementary Figure 10 | Sublimation of a pT nanocrystal at room temperature. a, AFM images of a nanocrystal sublimating at room temperature at times t_s = 0 h, 11 h, 16 h, and 21 h after printing. The scale bar is 200 nm. **b,** Change in lateral and vertical dimensions and the crystal volume over time associated with the sublimation of the crystal.

Supplementary Note 2: Molecular dipole orientation and crystallinity of printed pT

The crystallinity of the printed structures can be assessed indirectly by measuring the orientation of the molecules in the pT host matrix. In a crystalline host, terrylene is characteristically integrated in pT with a specific orientation given by the host crystal structure[10]. Therefore, the orientation of terrylene molecules in pT films is a very good indirect indicator for its crystallinity. Supplementary Fig. 11 shows a histogram of measured angular orientation of the spin coated molecules in crystalline, thin film pT host. A Gaussian fit shows that the molecules dipole orientation is distributed around an angle of 17.4°. By comparing this to Fig. 2d, which shows a histogram of measured angular orientation of nanoprinted molecules, it is evident that the two histograms are very similar. This similarity indirectly confirms the crystallinity of the printed pT nanostructures. A Gaussian fit shows that nanoprinted molecules dipole orientation is distributed around an angle of 15.4°. This small discrepancy may have statistical origin (due to the limited number of measured molecules) or could have a physical explanation, which would require much more quantitative studies. The high level of photostability of the molecules (see Supplementary Fig. 2 above) and the ability to withstand high illumination powers (see Supplementary Fig. 3 above) are other indirect indications of the crystallinity.

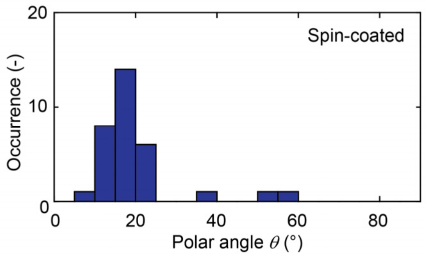

Supplementary Figure 11 | Angular orientation of spin coated molecules. Histogram of angular orientation of molecules embedded in a crystalline host matrix prepared by spin coating.

Supplementary Note 3: Probability analysis printing vs. stochastic placement

In many nano-optical experiments, one aims to place a single molecule within a few tens of nanometers of a certain structure such as nanoantennas, waveguides or microresonators. In stochastic distribution of molecules, one can only adjust the concentration of molecules in the solvent prior to spin coating or drop casting to vary the success rate of finding a molecule at the desired position. However, increasing the concentration also results in many molecules in the general vicinity and thus a large background fluorescence. The situation becomes increasingly more critical if one wishes to couple several molecules to a structure such as a waveguide or a microresonator by placing them at well-defined positions.

To point out the advantage of our printing method for such applications, we now analyze a concrete example, where individual molecules are to be placed in close proximity of a silver nanowire waveguide of length L. We define the coupling of the molecule to the wire to be successful if the molecule is placed within an area of A around the wire center and no other molecules are at a diffraction-limited spot with a radius r from the wire center, yielding an area $B = (L + 2r) \cdot 2r$.

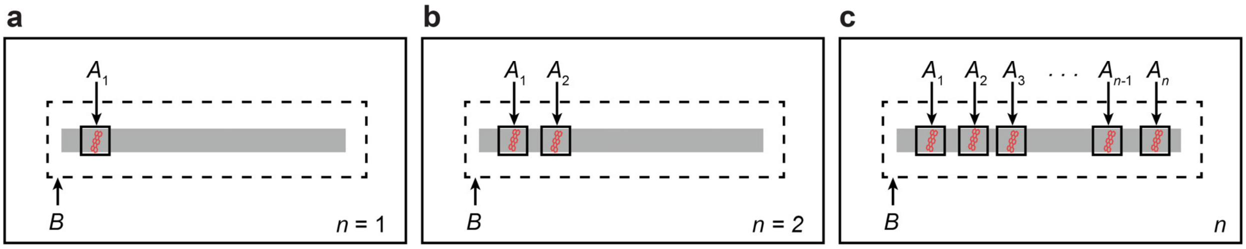

Supplementary Figure 12 | Probability analysis of coupling multiple emitters to a waveguide. a, Positioning a single molecule (n = 1) at a specific location on the waveguide within an area A_1 and an area of B without any molecule present. **b,** Positioning two molecules (n = 2) at specific, separate locations on the waveguide within areas A_1 and A_2. **c,** Positioning n molecules at specific, separate locations on the waveguide within areas A_n.

Placing the molecules by electrohydrodynamic printing has shown a probability 90% of depositing a molecule within ± 120 nm of the desired location (see Fig. 2e). Assuming that the

molecules are homogeneously distributed in the ink, the number *n* of printed molecules per spot at an optimally adjusted printing time (or ink concentration) follows a Poisson distribution with a maximum probability of $P(n) = e^{-n}\frac{n^n}{n!}$, thus the maximum probability of obtaining only a single molecule is *P(1)* = 0.368. This results in a probability of placing *n* molecules at *n* specific, separate locations (with ± 120 nm accuracy) of

$$P_{\mathrm{p}}(n) = (0.9 \cdot 0.368)^n \ . \tag{1}$$

For the numeric comparison we conveniently choose the area of placement *A* = 0.24 μm x 0.24 μm, which corresponds to the accuracy of ± 120 nm mentioned above, and for *L* = 10 μm and *r* = 0.2 μm yielding *B* = 10.4 μm x 0.4 μm.

Providing a quantitative estimate for stochastic coverage of a waveguide by molecules is not straightforward. We assume that one can adjust the concentration such that only one molecule is deposited within the area *B* on average. The maximum probability of this event can be obtained from a Poisson distribution and is 36.8%. The probability of placing the molecule in a specified subarea *A*, with no molecules in the remaining area *B - A* is thus obtained by $P_{\mathrm{S}}(1) = 0.368\frac{A}{B}$ (See Supplementary Fig. 12a). With the adjusted optimal concentration, the probability for placing two emitters at specified, separate locations is then obtained by $P_{\mathrm{S}}(2) = 0.368^2\left(\frac{A}{B}\right)^2 \cdot 2$ (See Supplementary Fig. 12b). Finally, for the general case of placing *n* molecules placed at *n* specified, separate locations this gives (See Supplementary Fig. 12c)

$$P_{\mathrm{S}}(n) = 0.368^n\left(\frac{A}{B}\right)^n n! \tag{2}$$

From Supplementary Eq. (1) and (2) the mean number of trials necessary for a successful outcome can be calculated and is given in Supplementary Table 2 for the example calculation.

**Number of emitters**	**1**	**2**	**3**	**4**
Printing	3	10	28	84
Stochastic	196	19,258	1,259,847	61,813,149

Supplementary Table 2 | Mean number of repetitions required for successful coupling of molecules to specific, separate locations on a waveguide. Numbers are given for the example calculation of an area A = 0.24 μm x 0.24 μm and B = 10.4 μm x 0.4 μm, considering a probability of 90% of printing a molecule within ± 120 nm of the desired spot.

The number of repetitions needed for a printing with higher accuracy, can be obtained by replacing the factor 0.9 in Supplementary Eq. (1) by the normalized yield shown in Fig. 2e. For coupling a molecule with an increased accuracy of ± 20 nm, a total of 28 repetitions are needed based on the normalized yield of 10%. Achieving this accuracy with random placement requires 706,522 repetitions according to Supplementary Eq. (2).

Supplementary Note 4: Printing on silver nanowires

Coupling quantum emitters to silver nanowires has been extensively studied with random placement methods[11] or lithographic methods[12]. Here we explore the feasibility of deterministically nanoprinting single molecules close to silver nanowires for coupling their fluorescence to the plasmonic mode of the nanowire. The coupling of quantum emitters to the surface plasmon polariton mode of a nanowire manifests itself in the out-coupling of fluorescence at the wire end, a lifetime shortening of the emitter and spectral modifications of the quantum emitter spectrum[13]. Supplementary Fig. 13a shows the fluorescence image of a molecule printed on a silver nanowire scaled to the maximum intensity and with an adapted intensity scale (7.8x) in order to observe the fluorescence out-coupling. Supplementary Fig. 13b shows the fluorescence image of the same nanowire after photobleaching of the molecule with the same intensity scalings as in Supplementary Fig. 13a. Supplementary Fig. 13c shows a white light image for localizing the nanowire. The out-coupling at the end of the nanowire, and moreover, the absence of out-coupling after the photobleaching of the molecule suggests that the molecule fluorescence is indeed coupled to the wire. By integrating the fluorescence intensity around the molecule, I_{m}, and around the wire end, I_{nw}, in the areas I and II shown with dashed red lines in Supplementary Fig. 13a, the apparent coupling efficiency is approximated as $\eta_{\mathrm{m}} = I_{\mathrm{nw}} / (I_{\mathrm{m}} + I_{\mathrm{nw}}) = 0.032$, not accounting for the ohmic losses in the nanowire (which accounting them would increase the effective coupling efficiency). Assuming a symmetric coupling of the fluorescence to both sides of the wire, an overall coupling of 6.2% of the molecule fluorescence to the plasmonic mode of the nanowire is estimated. By accounting for the ohmic losses with the absorption length L_{abs}, the actual coupling efficiency can be more accurately estimated with

$$\eta = \frac{e^{L/L_{\mathrm{abs}}} I_{\mathrm{nw}}}{I_{\mathrm{m}} + e^{L/L_{\mathrm{abs}}} I_{\mathrm{nw}}} \tag{3}$$

where L is the distance from the molecule to the end of the nanowire[11]. With an absorption length ranging between 2.7–3.7 μm as determined in literature for nanowires of similar diameter[11,14], this amounts to up to a factor of 3 to 5 when calculating the actual coupling efficiency. Similarly to previous studies we have assumed that light from the molecule and the wire end are collected by our oil objective with the same efficiency[11]. The background correction for the integrated intensities in Supplementary Eq. (3) is performed by subtracting the image after the photobleaching of the molecule.

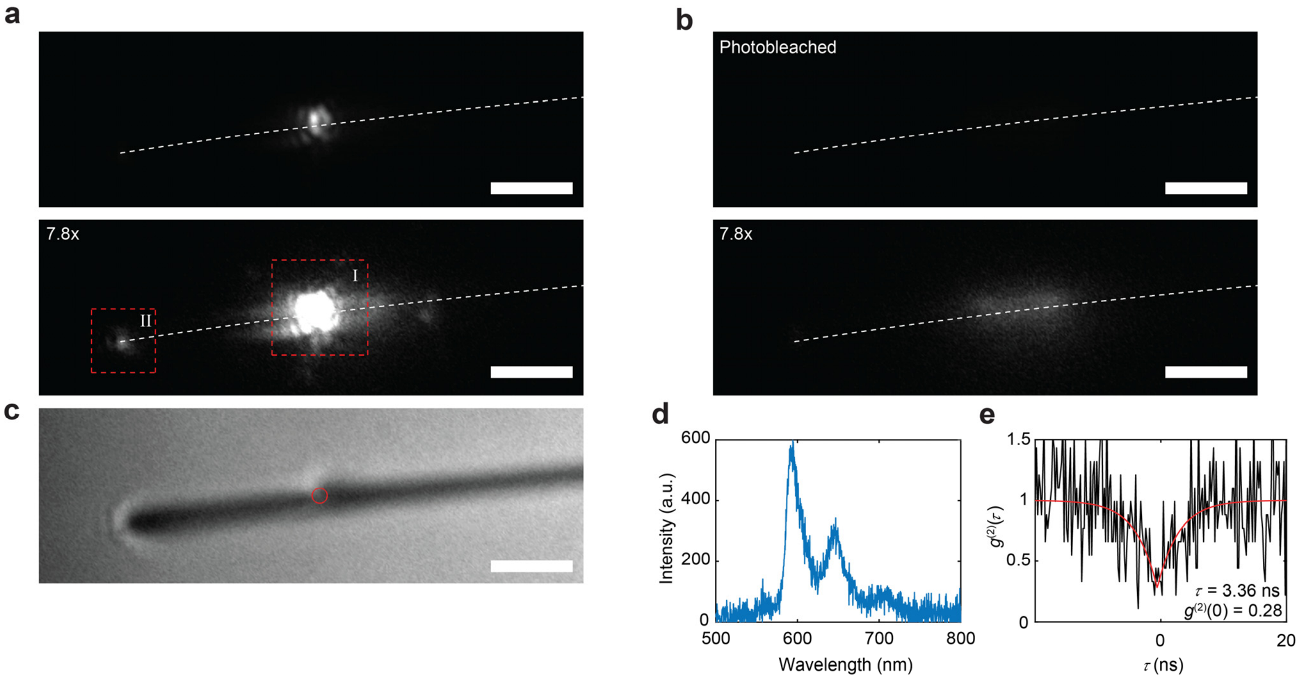

Supplementary Figure 13 | Coupling molecular fluorescence to a silver nanowire. a, Fluorescence images of a molecule printed on a silver nanowire scaled to the maximum intensity and with adapted intensity by a factor of 7.8. The dashed white line indicates the center of the nanowire. The dashed red squares indicate the areas for integrating the intensity for the efficiency calculation. **b,** Fluorescence images after photobleaching of the molecule scaled to the same intensities as in **a**. **c,** A white light image of the nanowire with the printed spot marked by a red circle. **d,** Fluorescence spectrum of the molecule printed on top of the wire. **e,** Second order correlation function measured from the molecule printed on top of the wire showing a lifetime of τ = 3.36 ± 0.8 ns and $g^{(2)}(0)$ = 0.28. The scale bars are 2 μm.

The fluorescence spectrum shown in Supplementary Fig. 13d is obtained from area I and verifies that the coupling is indeed from a terrylene molecule in proximity of the nanowire. Furthermore, the anti-bunching curve shown in Supplementary Fig. 13e, obtained from area I, verifies that only a single molecule is printed in area I and is coupled to the wire, and its lifetime is shortened to 3.36 ns by the presence of the wire (compare this lifetime with the lifetimes reported in Supplementary Fig. 16 below). To show the reproducibility of the method, a similar case is also shown in Supplementary Fig. 14a-d where an apparent coupling efficiency of 4.6% and a lifetime of 2.15 ns are obtained.

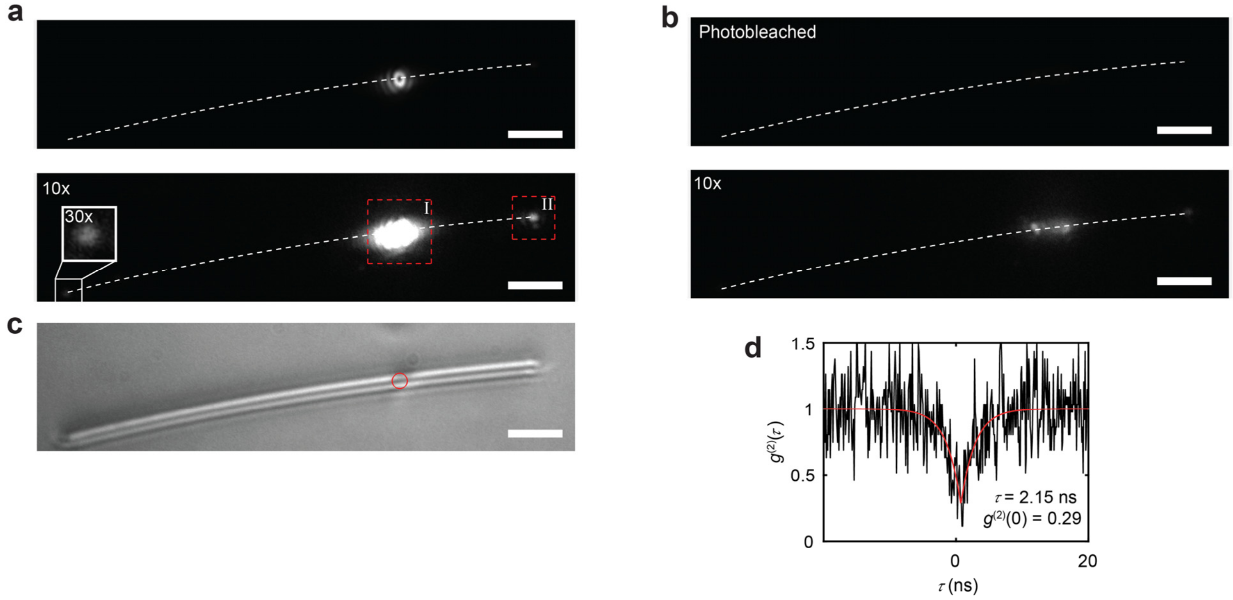

Supplementary Figure 14 | Coupling molecular fluorescence to a silver nanowire. a, Fluorescence images of a molecule printed on a silver nanowire scaled to the maximum intensity and with adapted intensity by a factor of 10. The dashed white line indicates the center of the nanowire. The dashed red squares indicate the areas for integrating the intensity for the efficiency calculation. The inset shows the faint fluorescence signal form the further wire end with an adapted intensity by a factor of 30 and magnified by a factor of 2 for better visibility.
b, Fluorescence images after photobleaching of the molecule scaled to the same intensities as in **a**. **c,** A white light image of the nanowire with the printed spot marked by a red circle. **d,** Second order correlation function measured from the molecule printed on top of the wire showing a lifetime of τ = 2.15 ± 0.3 ns and $g^{(2)}(0)$ = 0.29. The scale bars are 2 μm.

In the case of an intermittent, blinking molecule coupled to the wire, a strong temporal correlation between the molecule emission and the nanowire out-coupling is observed. Supplementary Fig. 15 shows a blinking molecule on top of a nanowire and the correlated out-coupling at the wire end.

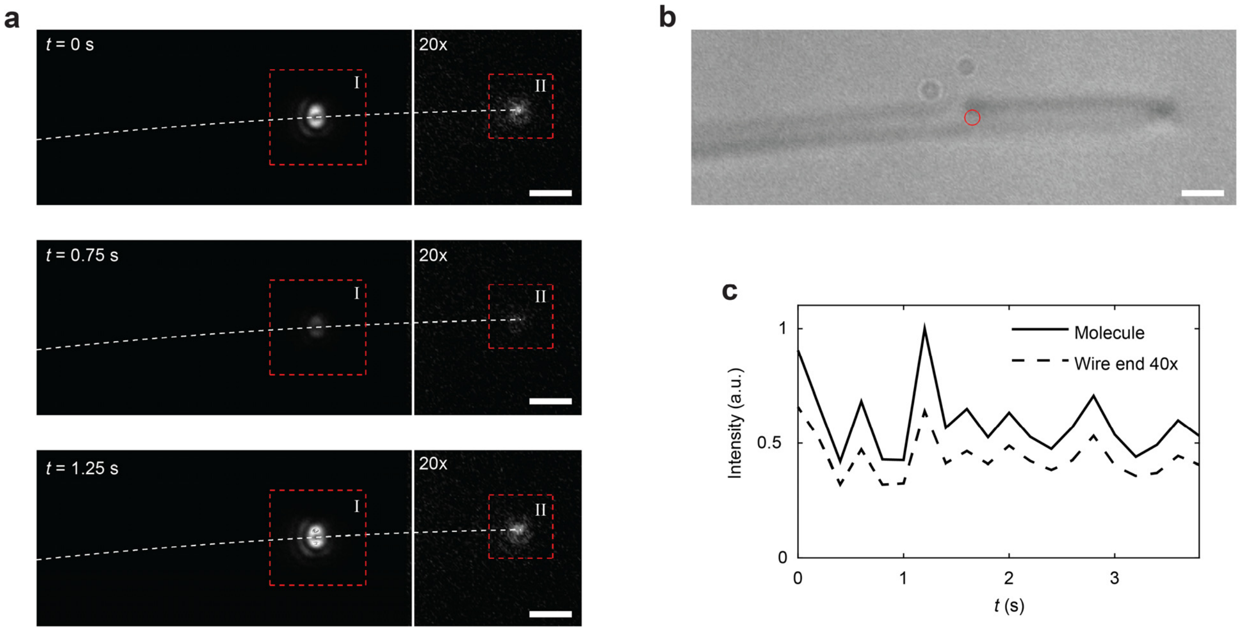

Supplementary Figure 15 | Correlated intensity of the molecule and nanowire end. a, Fluorescence images of a molecule printed on a silver nanowire at time t = 0, 0.75 and 1.25 s, scaled to the maximum intensity in the left part and in the right part of the image with adapted intensity by a factor of 20 for better visibility. The dashed white line indicates the center of the nanowire. The dashed red squares indicate the areas for integrating the intensity for the intensity calculation in (c) and efficiency calculation. **b,** A white light image of the nanowire with the printed spot marked by a red circle. **c,** Fluorescence intensity from the molecule and the nanowire end over time.

Supplementary Fig. 16 shows a histogram of the fluorescence lifetimes of molecules coupled to nanowires and the lifetimes of uncoupled molecules for comparison. With an average lifetime of 2.6 ns, we observe a lifetime shortening by a factor of 3.4 compared to the uncoupled average lifetime.

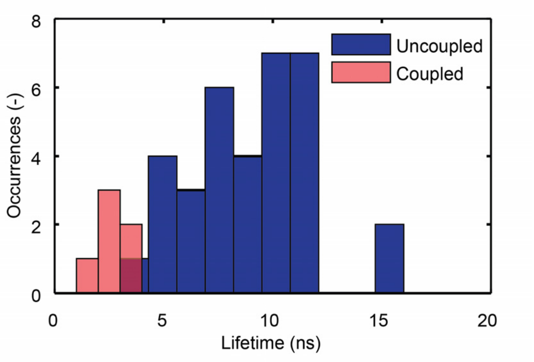

Supplementary Figure 16 | Distribution of fluorescence lifetime of coupled and uncoupled molecules. A histogram of measured fluorescence lifetimes of molecules coupled to a single nanowire and of uncoupled molecules. Fluorescence lifetimes are determined by fitting a single exponential to measured anti-bunching curves.

The size of the nanowires was measured from scanning electron microscopy (SEM) images. Supplementary Fig. 17 shows an image of a nanowire and the distribution of the nanowire diameter as measured from SEM images taken from several wires.

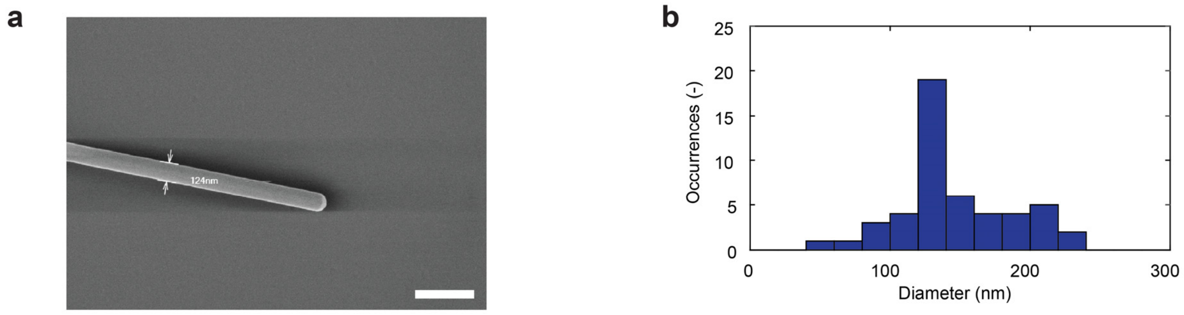

Supplementary Figure 17 | Nanowire diameter distribution. a, Scanning electron micrograph of a silver nanowire used for the molecule nanowire coupling experiment. The scale bar is 400 nm. **b,** Distribution of the nanowire diameters as measured from SEM images taken from several wires.

To provide further insight into the coupling of molecular emission to waveguides, we numerically analyzed the dependence of coupling efficiency on the position of the molecule

with respect to the silver nanowire. Supplementary Fig. 18 shows the calculated coupling efficiency (a) and Purcell factor (b) for a molecule in the pT crystal coupled to a silver nanowire at different positions in the crystal with respect to the waveguide. The results are only shown within the confined crystal (its border shown with the solid line). The radius of the nanowire and the dimensions of the pT host were obtained from atomic force microscopy scans. From experiment, a coupling efficiency ranging between 18–30% and a Purcell factor ranging between 1.8–4.5 were determined. The large variation in Purcell factor is due to the large measured variation in lifetime of the uncoupled molecules (see Supplementary Fig. 16). In Supplementary Fig. 18a the area shades in white corresponds to the locations where the simulated coupling efficiency is below 30%. In Supplementary Fig. 18b the area shaded in white correspond to the locations where the Purcell factor is below 4.5. Combining these two areas in Supplementary Fig. 18c gives the position of the molecule that is in accordance with the measurement results, highlighted in red. Based on these results and assuming that the molecule is on top of the nanowire, we estimate a distance of 45–70 nm between the molecule and the nanowire.

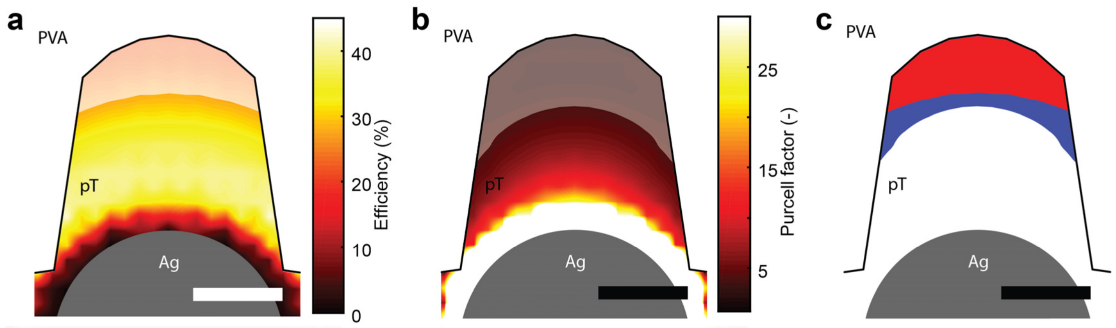

Supplementary Figure 18 | Molecule-nanowire distance estimation. Simulated coupling efficiency **a,** and Purcell factor **b,** depending on the location of the molecule with respect to the silver nanowire. Calculations were performed for a vertically oriented dipole inside the printed pT nanocrystal (with its border shown with the solid black lines) and a nanowire of 80 nm diameter. The size of the nanowire and host are approximated form AFM scans on the PVA coated structure. The white shaded regions indicate the possible position of the molecule.

c, Combining the possible locations from **a,** and **b,** gives the area marked in red, which highlights the possible locations of the molecule in the crystal that are in accordance with experimental results. The scale bars are 30 nm.

The above shown numerical estimations of the position of the molecules carry different uncertainties. One contribution to this is the orientation of the emitter, which is not known from the measurement. Furthermore, the exact diameter of the nanowire is difficult to assess as well as its dielectric function. The exact distance between molecule and silver nanowire is therefore difficult to determine.

Supplementary Note 5: Printing on a dielectric waveguide

Coupling molecular quantum emitters to dielectric waveguides has been studied with random placement methods[15,16]. Here we deterministically position single molecules in close vicinity of dielectric waveguides for coupling their fluorescence to the guided mode. Supplementary Fig. 19a shows the fluorescence image of molecules printed in close proximity of a dielectric TiO_2 waveguide scaled to the maximum intensity and with an adapted intensity scale (5x) in order to observe the fluorescence out-coupling. Supplementary Fig. 19b shows the fluorescence image of the same waveguide after photobleaching of the molecule on top of the waveguide with the same intensity scaling as in Supplementary Fig. 19a. Supplementary Fig. 19c shows a white light image of the waveguide and grating couplers. The out-coupling at the grating couplers, and the absence of out-coupling after bleaching of the molecule suggests that the molecule fluorescence is indeed coupled to the guided mode. As done in previous work[16], we estimate the emitter-waveguide coupling efficiency β based on the fluorescence intensity of the molecule I_m and the grating couplers I_c using the collection efficiency of the imaging objective η_o and the grating coupler efficiency η_c. The emitter waveguide coupling efficiency is then obtained by

$$\beta = \frac{I_c / \eta_c}{I_c / \eta_c + I_m / \eta_o} \tag{4}$$

The collection efficiency of the imaging objective is estimated to be η_o = 12 ± 2% from finite-difference time-domain (FDTD) simulations of a Herzian dipole on top of a TiO_2 waveguide covered with a 100 nm thick PVA layer. Based on this analysis we estimate β = 12 ± 2% with a grating coupler efficiency of 43%[15]. The background correction is performed by subtracting the image after the bleaching of the molecule from the raw image.

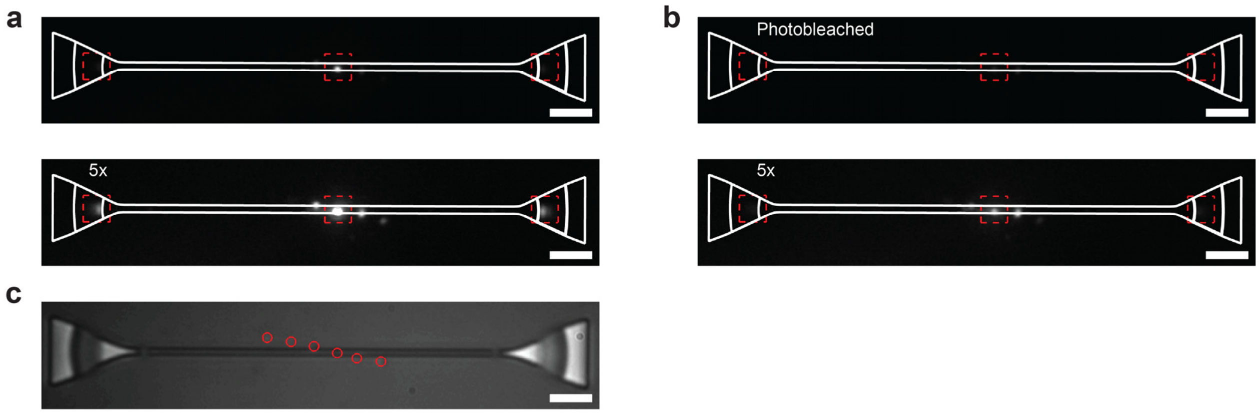

Supplementary Figure 19 | Coupling molecular fluorescence to a dielectric waveguide. a, Fluorescence images of a molecule printed on a dielectric TiO_2 waveguide scaled to the maximum intensity and with adapted intensity by a factor of 5. The solid white line indicates the borders of the waveguide and the grating couplers. The dashed red squares indicate the areas for integrating the intensity for the efficiency calculation. **b,** Fluorescence images after photobleaching of the molecule scaled to the same intensities as in (a). **c,** A white light image of the waveguide and grating couplers with the printed spots marked by red circles. The scale bars are 4 μm.

Supplementary References

1. Lindfors, K., Kalkbrenner, T., Stoller, P. & Sandoghdar, V. Detection and spectroscopy of gold nanoparticles using supercontinuum white light confocal microscopy. *Phys. Rev. Lett.* **93,** 037401 (2004).
2. Mortensen, K. I., Churchman, L. S., Spudich, J. A. & Flyvbjerg, H. Optimized localization analysis for single-molecule tracking and super-resolution microscopy. *Nat. Methods* **7,** 377–381 (2010).
3. Santhosh, K., Bitton, O., Chuntonov, L. & Haran, G. Vacuum Rabi splitting in a plasmonic cavity at the single quantum emitter limit. *Nat. Commun.* **7,** 11823 (2016).
4. Toyli, D. M., Weis, C. D., Fuchs, G. D., Schenkel, T. & Awschalom, D. D. Chip-scale nanofabrication of single spins and spin arrays in diamond. *Nano Lett.* **10,** 3168–3172 (2010).
5. Funke, J. J. & Dietz, H. Placing molecules with Bohr radius resolution using DNA

origami. *Nat. Nanotechnol.* **11,** 47–52 (2016).

6. Gopinath, A., Miyazono, E., Faraon, A. & Rothemund, P. W. K. Engineering and mapping nanocavity emission via precision placement of DNA origami. *Nature* **535,** 401–405 (2016).

7. Chen, Y. C. *et al.* Laser writing of coherent colour centres in diamond. *Nat. Photonics* **11,** 77–80 (2016).

8. Chen, Y.-C. *et al.* Laser writing of individual atomic defects in a crystal with near-unity yield. Preprint at http://*arXiv.org/abs/1807.04028* (2018).

9. Sapienza, L., Davanço, M., Badolato, A. & Srinivasan, K. Nanoscale optical positioning of single quantum dots for bright and pure single-photon emission. *Nat. Commun.* **6,** 8833 (2015).

10. Pfab, R. J. *et al.* Aligned terrylene molecules in a spin-coated ultrathin crystalline film of p-terphenyl. *Chem. Phys. Lett.* **387,** 490–495 (2004).

11. Akimov, A. V. *et al.* Generation of single optical plasmons in metallic nanowires coupled to quantum dots. *Nature* **450,** 402–406 (2007).

12. Gruber, C., Kusar, P., Hohenau, A. & Krenn, J. R. Controlled addressing of quantum dots by nanowire plasmons. *Appl. Phys. Lett.* **100,** 1–4 (2012).

13. Gruber, C., Trügler, A., Hohenau, A., Hohenester, U. & Krenn, J. R. Spectral modifications and polarization dependent coupling in tailored assemblies of quantum dots and plasmonic nanowires. *Nano Lett.* **13,** 4257–4262 (2013).

14. De Torres, J., Ferrand, P., Colas Des Francs, G. & Wenger, J. Coupling Emitters and Silver Nanowires to Achieve Long-Range Plasmon-Mediated Fluorescence Energy Transfer. *ACS Nano* **10,** 3968–3976 (2016).

15. Türschmann, P. *et al.* Chip-based all-optical control of single molecules coherently coupled to a nanoguide. *Nano Lett.* **17,** 4941–4945 (2017).

16. Lombardi, P. *et al.* Photostable Molecules on Chip: Integrated Sources of Nonclassical Light. *ACS Photonics* **5,** 126–132 (2018).
